# “Taking action” to reduce pain—Has interpretation of the motor adaptation to pain been too simplistic?

**DOI:** 10.1371/journal.pone.0260715

**Published:** 2021-12-08

**Authors:** Michael Bergin, Kylie Tucker, Bill Vicenzino, Paul W. Hodges

**Affiliations:** 1 NHMRC Centre of Clinical Research Excellence in Spinal Pain, Injury and Health, School of Health and Rehabilitation Sciences, The University of Queensland, St Lucia, Australia; 2 Neurorehabilitation Laboratory, Spaulding Rehabilitation Hospital, Charlestown, MA, United States of America; 3 School of Biomedical Sciences, The University of Queensland, St Lucia, Australia; National Tsing Hua University, TAIWAN

## Abstract

Movement adapts during acute pain. This is assumed to reduce nociceptive input, but the interpretation may not be straightforward. We investigated whether movement adaptation during pain reflects a purposeful search for a less painful solution. Three groups of participants performed two blocks (Baseline, Experimental) of wrist movements in the radial-ulnar direction. For the Control group (n = 10) both blocks were painfree. In two groups, painful electrical stimulation was applied at the elbow in Experimental conditions when the wrist crossed radial-ulnar neutral. Different stimulus intensities were given for specific wrist angles in a secondary direction (flexion-extension) as the wrist passed radial-ulnar neutral (*Pain 5–1 group*:painful stimulation at ~5 or ~1/10—n = 21; *Pain 5–0 group*:~5 or 0(no stimulation)/10—n = 6)). Participants were not informed about the less painful alternative and could use any strategy. We recorded the percentage of movements using the wrist flexion/extension alignment that evoked the lower intensity noxious stimulus, movement variability, and change in wrist/forearm alignment during pain. Participants adapted their strategy of wrist movement during pain provocation and reported less pain over time. Three adaptations of wrist movement were observed; (i) greater use of the wrist alignment with no/less noxious input (Pain 5–1, n = 8/21; Pain 5–0, n = 2/6); (ii) small (n = 9/21; n = 3/6) or (iii) large (n = 4/21; n = 1/6) change of wrist/forearm alignment to a region that was not allocated to provide an actual reduction in noxious stimulus. Pain reduction was achieved with “taking action” to relieve pain and did not depend on reduced noxious stimulus.

## Introduction

Theories of the motor adaptation to pain [1–4] posit that movement is altered by the nervous system as a purposeful attempt to reduce nociceptive input and pain, and to protect structures (e.g. muscle, ligament) from further injury. Movement may adapt in several ways, such as reduced movement amplitude [5, 6], reduced velocity [6], reduced force [7], modified force direction [8, 9], altered movement variability [10–12], removal of the body part from the painful situation [13], or complete avoidance of a task [14]. Although the purpose of the adaptation appears straightforward–to reduce pain by reducing nociceptive input–it has not been tested: *whether* nociceptive input is reduced in the adapted movement solution; *why* a particular movement strategy is selected (e.g. selection of a specific direction of knee extension force during acute pain [9]) from the many options that are available; and *how* the search is conducted.

Whether motor adaptation during pain reduces nociceptive input remains unclear. Although modified movement can reduce the subjective report of pain [15], this does not confirm reduced nociception. This is because pain and nociception are not linearly related. Properties such as placebo provide evidence of multifactorial nature of the relationship. Whereas nociception involves activation of the peripheral nociceptive neurons, pain is an experience that depends on interpretation of nociceptive input, biological processes that modulate input, as well as multiple cognitive/psychological factors [16]. It has been argued that “taking action” (i.e. choosing a new movement strategy with the intention to reduce pain/threat) is sufficient to reduce the experience of pain, despite no change in nociceptive input [17]. It is not yet clear whether reduced excitation of peripheral nociceptors is necessary to achieve pain relief from an adapted movement strategy during a painful movement.

Reduced noxious input may not be the only factor considered when selecting an adaptation. It is plausible that other factors may influence the final solution. For instance, energy “cost” may be considered; if the new solution involves greater energy demand it may not be selected despite a benefit of reduced nociceptive input [18]. Here we tested whether participants adopted a movement strategy selected externally by the experimenter to involve less noxious input, or whether they selected another solution.

It has been proposed the nervous system undertakes a purposeful search for a less painful strategy by experimenting with different movement patterns (i.e. various combinations of motion of body segments/joints and muscle activity; “elements”) to reduce pain and/or nociceptive input, and may take advantage of exposure to alternative options through between-repetition variability of these elements in that search [1, 11, 12]. Although plausible, and there is some evidence that movement variability increases during acute pain in complex tasks that involve multiple elements that can be varied while still maintaining task success [11, 12], it remains unclear whether the variation constitutes a purposeful search. It might instead reflect pain’s interference with movement control [19]. Here we challenge the interpretation of variability as a search for a new solution by investigation of the between-trial variation and changes in movement strategy in individual participants during exposure to a painful movement.

The aims of this study were to investigate critical questions in the understanding of motor adaptation to pain. First, we aimed to resolve whether reduced pain depends on reduced excitation of nociceptive afferents. Second, we tested whether adaptation involves modification to the solution that achieves the greatest reduction of excitation of nociceptive afferents. Third, we challenged whether increased movement variability facilitates the search for a new movement strategy.

## Materials & methods

### Study design

**Fig 1:**
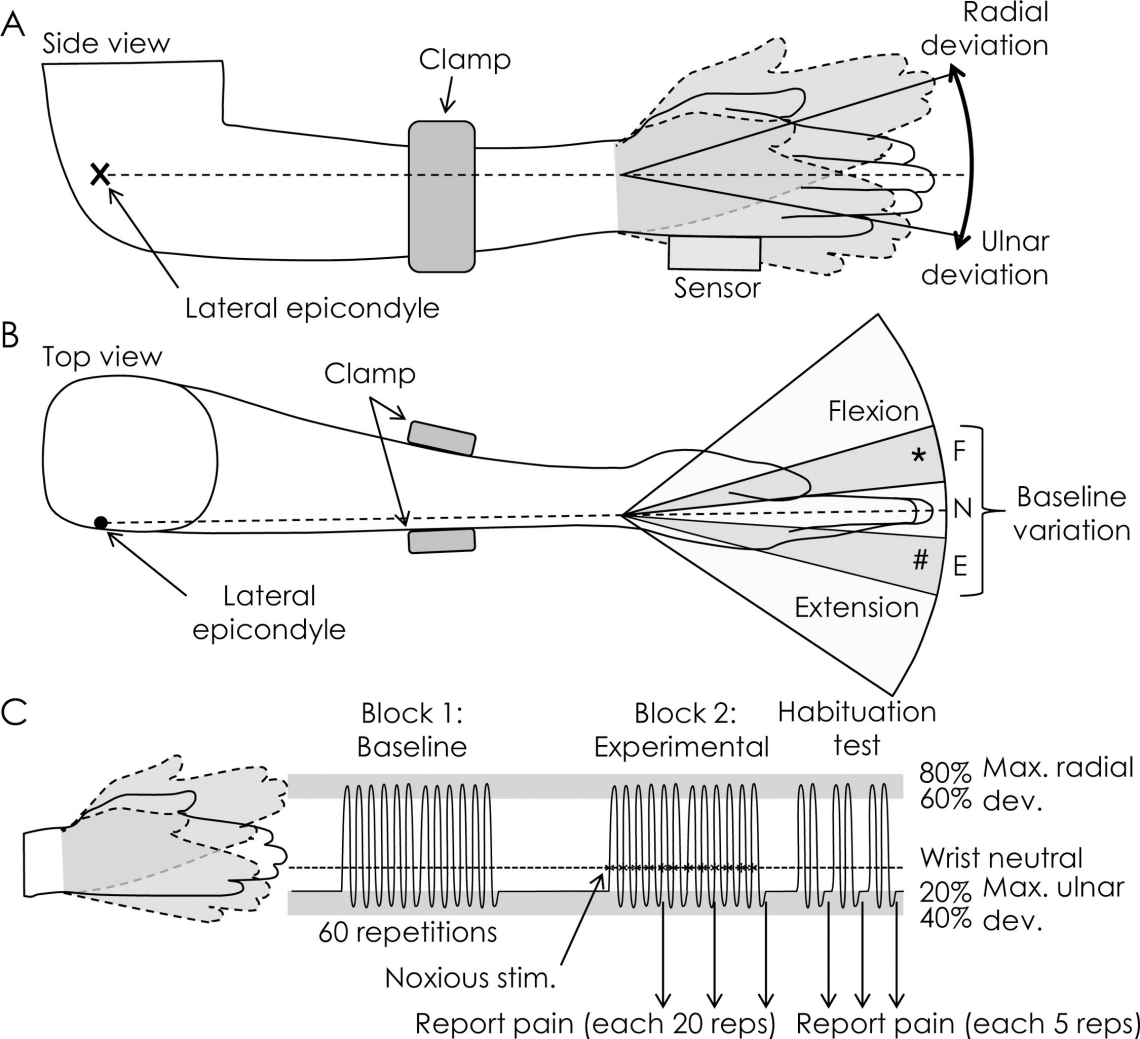
The experimental task involved repetitive radial-ulnar deviation movements between target regions in each direction. In the *Pain 5–1* and *Pain 5–0* experiments less or no, respectively, noxious stimulation was determined by the alignment of the wrist in the flexion-extension plane (B) as the wrist passed through radial-ulnar deviation neutral (A). The range of variation in the flexion and extension direction was identified in the Baseline condition and one third of this range was selected either in the flexion (*) or extension (#) direction. Movements performed with alignment in the flexion-extension plane in this target region either induced no stimuli or stimuli with reduced intensity. Panel C shows the timeline of the experiment. Max–maximum, dev–deviation, stim–stimulation, reps–repetitions.

To investigate these questions we studied a standardized task (Fig 1) that required wrist radial-ulnar deviation movement between two target regions for multiple repetitions. This task “goal” in the radial/ulnar direction can be achieved, but with variation in the alignment of the wrist/forearm in the other movement planes (flexion-extension, pronation-supination) [10]. An experimental paradigm was developed whereby a noxious stimulus (in most repetitions) was applied to the elbow during radial-ulnar deviation task (primary movement plane) as the wrist past through the neutral position in that plane. The painful stimulus was moderately painful (target pain– 5/10 on a 11-point numerical rating scale anchored with no pain at zero and worst pain imaginable at 10) in most repetitions. Without the knowledge of the participants, in some repetitions, lower intensity or no stimuli were provided to induce less pain (target pain– 1/10; *Pain 5–1* group) or no pain (target pain– 0/10; *Pain 5–0* group). This lower intensity or no stimulus was provided if the radial-ulnar movement was performed with a specific alignment of the wrist in the flexion-extension plane (secondary plane). We measured the natural range of variation in the flexion-extension plane that would be used by a participant to ensure that the alignment in that plane used to induce the less intense noxious stimulus was within the range the participant would normally experience. We hypothesized that: (i) at the end of a block of repetitions with noxious stimulation, participants would report less pain than at the start of the block, which we presumed would be the result of identification of a less provocative movement strategy; (ii) participants would continue to successfully achieve the goal to attain the target radial deviation angle during the painful block of repetitions; (iii) participants would modify motion in secondary planes during the primary movement; (iv) if a substantially less painful solution was provided by the experimental paradigm, and was experienced by the participant by chance as a result of changing their movement strategy, this less painful strategy would be selected more frequently than other options for movement and participants would report less pain at the end of the block of repetitions, and (v) movement variability would initially increase such that a variety of movement options would be trialled in the search for a new less painful solution.

### Participants

Twenty-one volunteers (11 females; age 24 ± 6 years) were included in the *Pain 5–1* group with noxious stimuli provided during the task at 5 and 1/10. Due to the novelty of the paradigm no data were available to estimate a sample size and a priori it was decided to include at least 20 volunteers. Ten volunteers (6 females; age 28 ± 4 years (mean ± SD)) were recruited for a *Control* group who performed the task with no noxious stimuli to assess the effect of time. Six volunteers (4 females; age 22 ± 4 years) were recruited for an additional control experiment (*Pain 5–0* group) in which noxious stimuli were provide at 5, and no stimuli (i.e., 0/10) were provided if the specified alignment in the second plane was achieved. This was included to test whether results would be similar when the reward for selecting the target region was complete rather than partial pain relief. All participants were naïve to the hypotheses of the study. Participants were excluded if they reported any major circulatory, orthopaedic, musculoskeletal or neurological conditions that affected upper limb function. All procedures were approved by the Institutional Medical Research Ethics Committee and participants provided written informed consent.

### Procedures

Participants sat upright with their right forearm resting on a table and supported in the mid-position between pronation and supination with the elbow in ~90° flexion (Fig 1). The forearm was secured with an adjustable clamp applied to the mid region of the forearm. The rig allowed unconstrained wrist motion but prevented movements of the proximal upper limb that could affect performance of the radial-ulnar deviation task. The clamp reduced but did not prevent pronation-supination of the forearm.

A motion sensor (SK7 SHAKE, SNMH Engineering Services, Ireland) was attached to the ulnar border of the right hand to measure radial-ulnar deviation and flexion-extension of the wrist, and forearm pronation-supination. The motion sensor signal was recorded at 100 samples per second using a data acquisition system (PCI-6035E, National Instruments, TX, USA) and Matlab 7.14 (The Mathworks, Natick, MA, USA). Motion of the sensor recorded motion with respect to the external reference frame, and was interpreted as wrist angle change.

Prior to the experimental blocks, the neutral position of the wrist and forearm, and the maximal range of motion for radial and ulnar deviation, were recorded. The range of wrist motion was measured using a handheld goniometer from the neutral position with the wrist and forearm in the mid position of flexion and extension, radial and ulnar deviation, and pronation and supination. This range was used to calculate and set movement targets.

The experimental task involved repeated radial-ulnar deviation of the wrist between two target angle regions that were displayed along with angle data on a computer monitor positioned ~60cm in front of the participant. Participants were instructed to move from a target angle region between 20–40% of their maximal ulnar deviation range to a target angle region between 60–80% of their maximal radial deviation range in time with a metronome set to 90 beats per minute (one movement = movement from ulnar to radial target and return to ulnar target). Emphasis was placed on movement to the target angle region in the radial deviation direction. Participants practiced the task at the start of the session until it was performed at the correct frequency with successful attainment of the ulnar and radial targets for ~10 repetitions. Two blocks (i.e. “Baseline” and “Experimental”) of sixty repetitions were recorded for each group. Each block started and finished with the wrist at the 20% ulnar deviation position, and neutral wrist flexion-extension and forearm pronation-supination. In the *Control* group the Baseline and Experimental blocks were identical.

### Noxious electrical stimulation of the elbow

**Fig 2:**
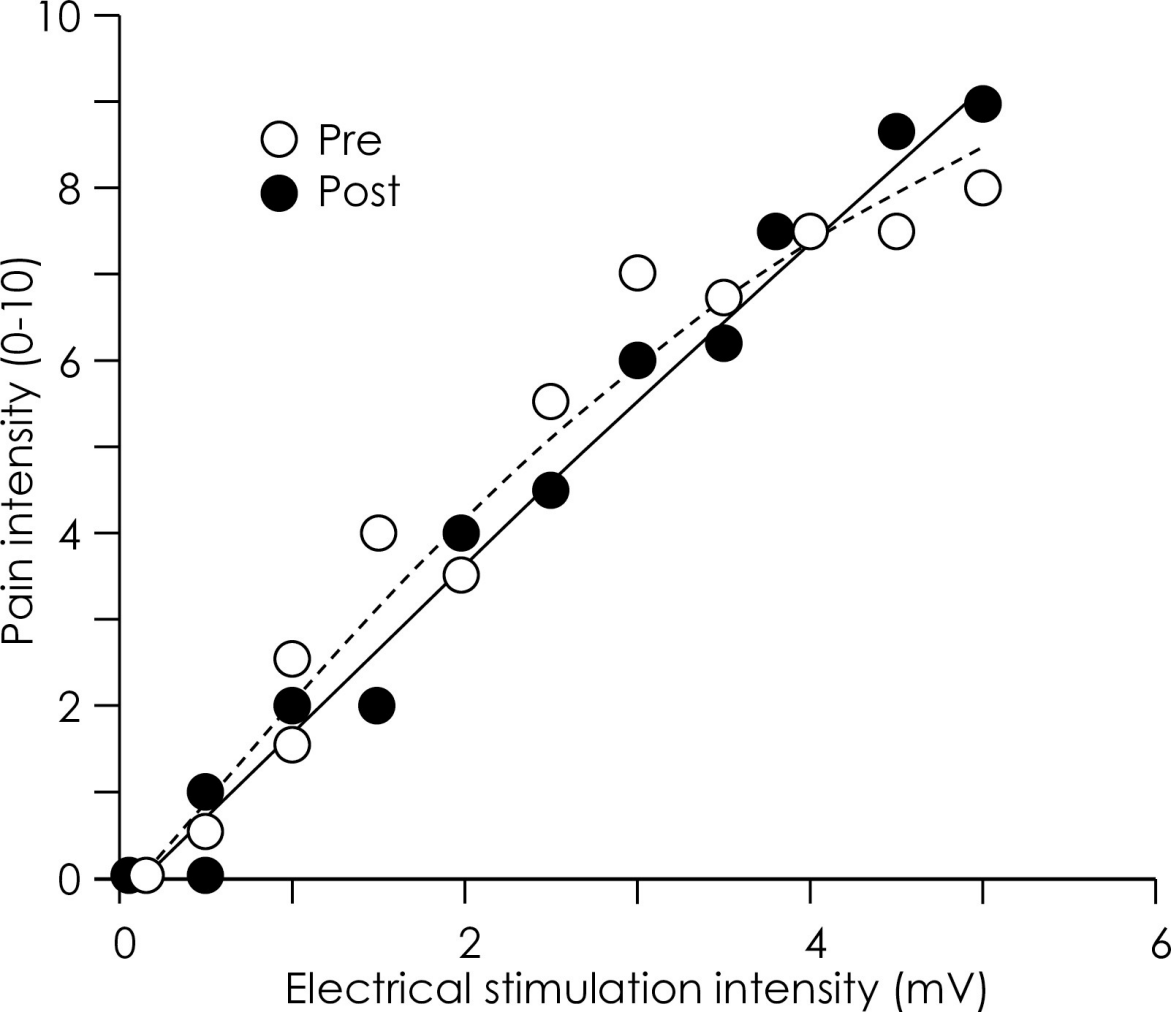
Representative data for the intensity of pain reported with electrical stimulation. Data are shown for pain rating (11-point numerical rating scale; 0–10) vs. stimulus intensity (0–10 mV) reported by a representative participant from the *Pain 5–1* experiment before (white circles) and after the baseline/experimental blocks (black circles). A quadratic function was fitted to the pre-movement data to determine the stimulus intensities required to elicit the desired pain intensity for the painful Experimental block.

Cutaneous electrical stimulation was applied to the elbow during the “Experimental” block of the *Pain 5–1* group (and the *Pain 5–0* group in the additional control experiment) to elicit nociceptive afferent excitation and pain. Stimuli were provided near the origin of the wrist muscles that induce extension and radial deviation (extensor carpi radialis longus) which approximates the region of pain in lateral epicondylagia (Fig 1A). Electrical stimulation has been used extensively for experimental induction of pain (e.g. [12, 20, 21]) as it permits application of a stimulus of known intensity and duration [22]. A pair of surface electrodes (inter-electrode distance ~10 mm; 3M, USA) was placed on the skin overlying the lateral epicondyle of the right elbow. The electrodes were placed over bone to avoid muscle contraction. Brief electrical stimuli (60Hz, 100-ms train, 1-ms pulse duration; Digitimer DS7A, UK) were applied with increasing intensity (0–10 mV; 1-mV increments) until participants verbally rated pain intensity of 8/10 on an 11-point numerical rating scale (NRS) anchored with ‘no pain’ at 0 and ‘worst pain imaginable’ at 10. A rating of 8/10 on the NRS was defined as the ‘maximum stimulus’ intensity for each participant. Twelve to fifteen stimuli of variable stimulus intensity (range: 0mV to ‘maximum stimulus’; order randomized) were then delivered to the elbow. Participants rated their pain on the NRS after each stimulus. The pain rating was plotted against the stimulus intensity and a quadratic function fitted to determine the stimulus intensities to be used to elicit the desired pain intensity for the painful blocks (Fig 2).

The experimental paradigm involved delivery of a moderately painful stimulus (~5/10 on the NRS) to the elbow during each repetition of wrist movement, but a less painful stimulus (~1/10 on the NRS: *Pain 5–1* group; no stimulus [0/10] for the additional control experiment: *Pain 5–0* group) was delivered if the participant used a radial-ulnar deviation movement strategy with a specific alignment in the wrist flexion-extension movement plane (Fig 1). Several steps were undertaken to specify the characteristics of the less (*Pain 5–1*) or non-painful (*Pain 5–0*) movement strategy. First, we identified the range of alignments of flexion/extension plane used naturally by a participant when moving from the ulnar deviation target to the radial deviation target. This involved measurement of the angle of the wrist in the flexion-extension plane as it passed through radial-ulnar neutral for each repetition of the “Baseline” block. The difference, in degrees, between the maximal wrist flexion and maximal wrist extension angles recorded during this Baseline block was defined as the ‘baseline flexion-extension range’ and divided into 3 equal regions (Fig 1B). In the “Experimental” block, painful electrical stimuli were applied to the elbow as the wrist crossed the neutral radial-ulnar deviation position. A region that corresponded to one third of the baseline flexion-extension range (randomly selected as one third of range towards the flexion or extension direction) was allocated for a less painful noxious stimulus (~1/10 on the NRS; *Pain 5–1*) or no stimulus (no pain; *Pain 5–0*) when the wrist crossed the neutral radial-ulnar deviation position (Fig 1). For radial-ulnar deviation movements performed with alignment in the remainder of the flexion-extension plane or alignments beyond the baseline flexion-extension range, the painful stimulus was applied at an intensity expected to evoke pain of 5/10 on the NRS. Participants were advised prior to the Experimental block that they “may or may not receive painful electrical stimuli as you perform the task” and were unaware that a less or non-painful movement strategy was available. After every 20 repetitions in the Experimental block, participants were asked to verbally rate the average pain they experienced over the preceding 20 repetitions using the NRS (Fig 1C).

Additional measurements were made after the completion of the Experimental block to determine whether habituation or sensitization to the noxious electrical stimuli developed during the blocks. Immediately after the completion of the Experimental block, participants performed 5 repetitions of the radial-ulnar deviation task within each flexion-extension region (n = 3) and either direction outside the ‘baseline flexion-extension range’ as electrical stimuli were delivered to the elbow as per the movement blocks (Fig 1C, habituation test). After each 5-repetition block the participants rated the intensity of pain (using the NRS) they had experienced for each region. We then delivered the same twelve to fifteen stimuli of variable stimulus intensity (range: 0 mV to ‘maximum stimulus’; order randomized) that were used at the start of the experiment and asked participants to rate their pain on the NRS after each stimulus. The pain rating was plotted against the stimulus intensity for each stimulus and a quadratic function was fitted to the data (Fig 2).

Absence of habituation or sensitization would be supported if: (i) the pain intensity reported during the start (i.e. repetitions 1–20) of the Experimental block was not different to the pain intensity recorded when participants performed 5 repetitions of the radial-ulnar deviation task within the moderately painful flexion-extension regions after completion of the Experimental block (Habituation test); and (ii) there was no difference in the stimulus intensities required to elicit 5/10 and 1/10 pain before and after the movement blocks.

### Data analysis and Statistical analysis

Data were analysed using Matlab (Mathworks, Nattick, USA). Statistical analysis was performed using SPSS Version 27 (IBM, USA). Significance was set at p<0.05 and effect size was calculated as partial eta squared for all ANOVA statistics. Sphericity was tested with Mauchly’s test and if violated, Greenhouse-Geisser correction was applied. Data are presented as mean (SD) throughout the text.

Pain intensity was compared for movements performed at the beginning (repetitions 1–20) and end (repetitions 41–60) of the Experimental block (repeated measure), and between Groups (*Pain 5–1* vs. *Pain 5–0*) using repeated measures analysis of variance (ANOVA) (Hypothesis i). Stimulus intensity required to evoke target pain amplitude, and the pain evoked by different stimulus intensities during movement were compared between blocks before and after the experiment with paired t-tests (two tails) to investigate habituation/sensitization to the noxious stimulus.

Successful attainment of the task goal was calculated as the percentage of repetitions (0–100%) within each block that were successfully terminated within the radial target angle region (Hypothesis ii). For the *Control* group, data are reported for 9 of 10 participants as the data for ‘successful attainment of the task goal’ for one participant was >2 standard deviations below the group mean and considered an outlier. Percentage of successful repetitions was compared between Blocks (Baseline vs. Experimental; repeated measure) and Groups (*Control* vs. *Pain 5–1* vs. *Pain 5–0;* between-subject factor) with repeated measures ANOVA. Post hoc testing was undertaken using Fisher’s least significant difference test for this and other analyses.

**Fig 3:**
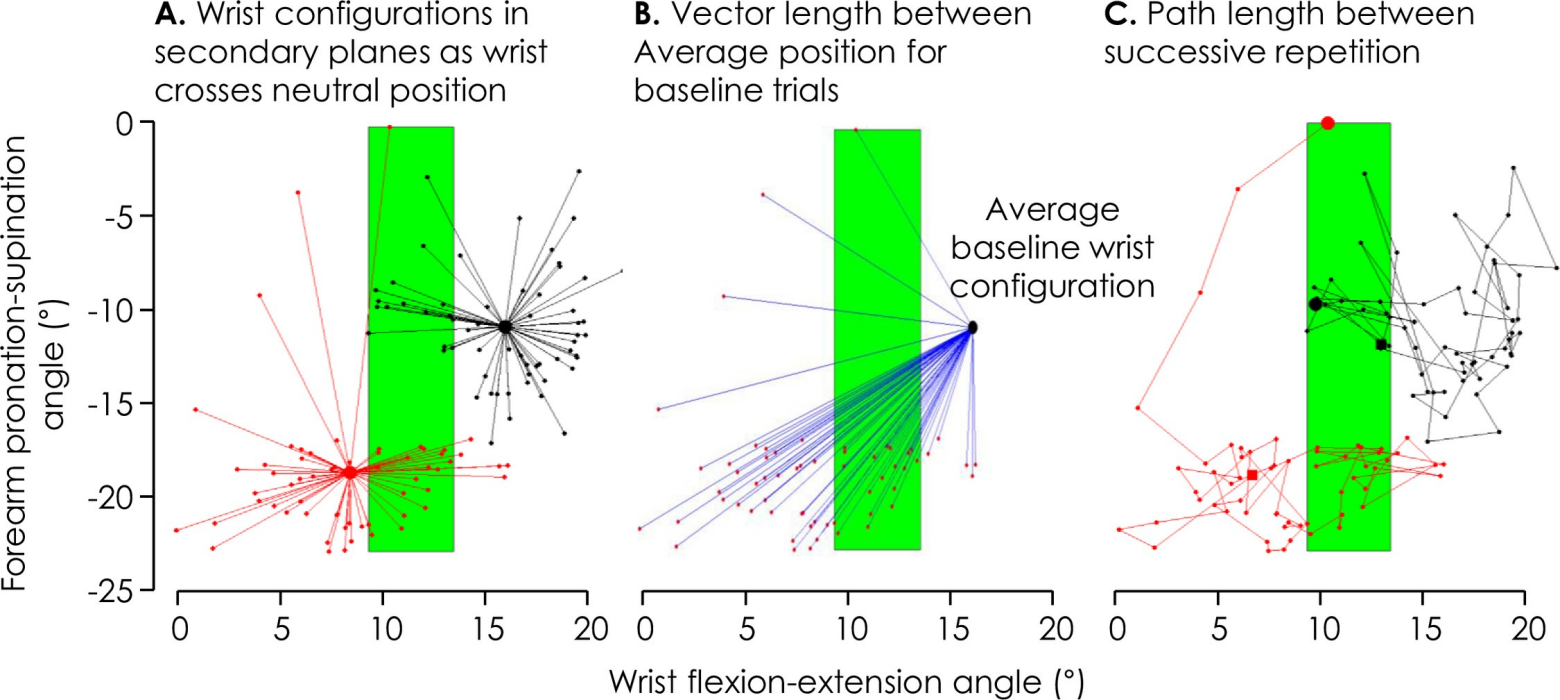
Three analysis measures demonstrated with data from a representative participant. **A.** Wrist configuration in the secondary planes (flexion-extension; pronation-supination) is shown for each repetition of movement at the moment the wrist crossed the neutral position in the primary plane (radial-ulnar deviation). Vector length is shown from the mean position in each direction for each entire block. Black–Baseline block; Red–Experimental block **B.** Vector length between the average configuration of the wrist at the moment it passed the neutral in the primary plane (radial-ulnar deviation) recorded for all repetitions in the Baseline block, and the position of the wrist for each repetition separately of the Experimental block. **C.** Path length between the wrist positions at the time it passed the neutral in the primary plane (radial-ulnar deviation) during successive repetitions for the Baseline and Experimental block. The large circle represents the start of the block and the box represents the end. In all panels the green box indicates the region of configurations designated to provide no/minimal noxious stimulation based on angle in the flexion-extension plane.

The angle of the wrist/forearm in flexion-extension and pronation-supination was calculated as the wrist passed through the neutral radial-ulnar deviation position when moving towards the radial target (Fig 1). To investigate whether wrist/forearm angle in these secondary planes was altered during the Experimental block relative to the Baseline block (Hypothesis iii), vectors were constructed using the *average* position of the wrist/forearm configuration (flexion-extension and pronation-supination position) of the Baseline block as the origin and the position of the wrist/forearm during each repetition of the Baseline and Experimental blocks as the end of the vector (Fig 3B). Although the pain stimulus was defined by the wrist position in the flexion-extension direction only, adaptation could occur in either of the secondary planes and it was considered necessary include both in the analysis. Each 60-repetition block was divided into 6 x 10-repetition epochs and the mean vector length calculated for each epoch. A change in position (i.e. adoption of a new strategy for wrist motion) would be identified as difference in vector length between Epochs (repeated measure) and between Groups (*Control* vs. *Pain 5–1* vs. *Pain 5–0;* between-subject factor) as identified with a repeated-measures ANOVA.

To compare the proportion of repetitions that adopted the “less/non-painful movement solution” (Hypothesis iv) we calculated the percentage of repetitions within each Block (Baseline and Experimental) in which the wrist crossed the neutral radial-ulnar deviation position with wrist alignment in the flexion-extension region that was designated for less/no noxious input. Percentages were compared between Blocks (Baseline vs. Experimental; repeated measure) and Groups (*Pain 5–1* vs. *Pain 5–0;* between-subject factor) with repeated measures ANOVA.

To quantify between-repetition variation of the elements of the movement (Hypothesis v), vectors were constructed between the wrist/forearm configuration of successive repetitions (e.g. 1–2, 2–3, …) in the Baseline and Experimental blocks (Fig 3). Each vector length represents the change in alignment in the secondary planes of the wrist/forearm between successive repetitions of radial-ulnar deviation. Each sixty-repetition block was divided into 6 x 10-repetition epochs and the sum of the path lengths calculated for each epoch. The sum of path lengths was compared between Blocks (Baseline vs. Experimental; repeated measure), Epochs (Epoch 1–6; repeated measure) and Groups (*Control* vs. *Pain 5–1* vs. *Pain 5–0;* between-subject factor) with repeated measures ANOVA.

## Results

### Did pain intensity change from the start to the end of the Experimental block?

Pain intensity was less for movement performed at the end than the start of the painful Experimental block (Main effect: Epoch: p = 0.01) for the *Pain 5–1* (start: 4.3(0.7); end: 3.2(0.7); mean pain reduction: 1.1(0.5)) and *Pain 5–0* (start: 4.3(1.1); end: 3.7(1.7); mean pain reduction: 0.6(1.7)) groups. We showed no evidence of habituation/sensitisation. The stimulus intensities required to elicit pain of 5/10 (pre: 3.3(0.7) mV; post: 2.8(0.5) mV; p = 0.31) and 1/10 (pre: 0.6(0.2) mV; post: 0.7(0.2) mV; p = 0.14) in the *Pain 5–1* group and 5/10 pain (pre: 3.2(1.3) mV; post: 3.7(1.6) mV; p = 0.15) in the *Pain 5–0* group did not differ between tests before and after the movement. Further, after completion of the Experimental block, participants rated pain as 4.4(0.4) when moving in moderate pain regions and 0.6(0.3) in the less painful region for the *Pain 5–1* group and 4.3(0.4) and 0.0(0.0), respectively, for the *Pain 5–0* group. Taken together, these data support our first hypothesis that during the Experimental block participants would find a less painful alternative to move by the end of the Experimental block and this was not explained by habituation to the noxious stimuli.

### Was attainment of the task goal affected by experimental elbow pain?

Contrary to our hypothesis (ii), participants did not maintain successful performance of the task during pain. Although the task goal was achieved consistently by the *Control* participants who did not experience pain (Baseline: 86(10)%; Experimental: 88(6)%; post hoc: p = 0.53), the goal was achieved less frequently (Interaction: Group×Block: F_2,34_ = 3.76, p = 0.03, η_p_^2^ = 0.19) during the Experimental block than Baseline for the *Pain 5–1* (Baseline: 90(3)%; Experimental: 80(6)%; post-hoc: p = 0.001) and *Pain 5–0* (Baseline: 95(3)%; Experimental: 82(9)%; post-hoc: p = 0.02) groups.

### Was out of plane movement changed during pain?

**Fig 4:**
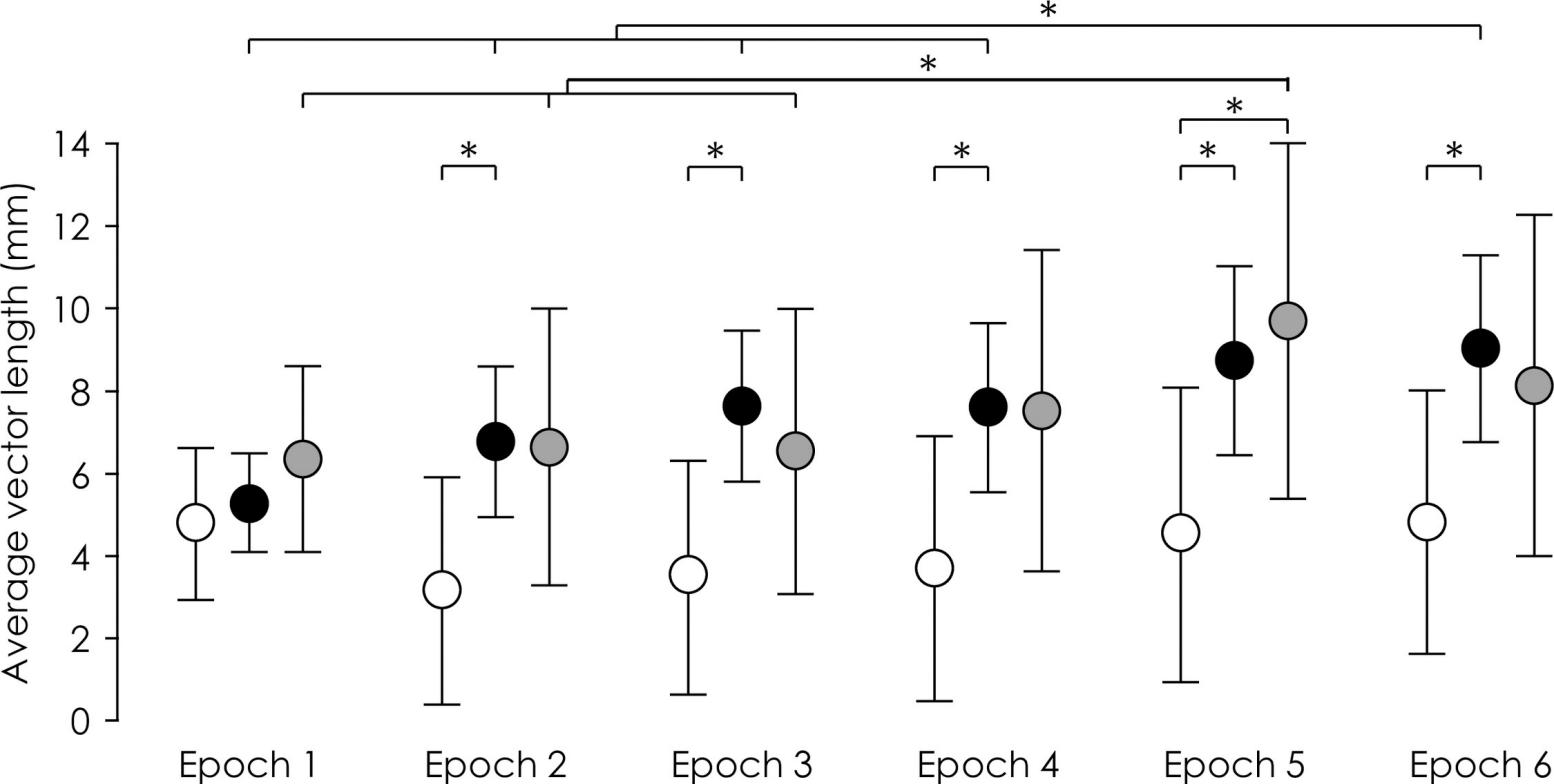
Group data for change of wrist/forearm alignment, quantified as average vector length relative to mean alignment used in Baseline blocks (refer to Fig 3B). Group mean and 95% CI of average vector length of 10-repetition epochs for the *Control* (white), *Pain 5–1* (black), and *Pain 5–0* (grey) participant groups are shown. *—p<0.05 between bracketed items.

In the *Control* group who did not receive noxious stimuli, the wrist/forearm alignment in the secondary planes, as measured by average vector length from the mean alignment of the wrist in the Baseline block, did not change between the initial and final epoch during the Experimental block (Interaction: Group×Epoch: F_10,170_ = 2.06, p = 0.03, η_p_^2^ = 0.12; post-hoc: all p>0.10; Fig 4). That is, the wrist/forearm alignment in the flexion-extension and pronation-supination directions remained consistent throughout repetitions for the *Control* group.

Although average vector length was initially (i.e., epoch 1) similar during the Experimental block of the three groups (post-hoc: p>0.50), during later epochs, average vector length was greater in the *Pain 5–1* than *Control* group for epochs 2–6 (post-hoc: all p<0.05), and greater at the end (epoch 6) than start (epochs 1–4) of the Experimental block (post-hoc: all p<0.04). That is, consistent with our hypothesis (iii), wrist/forearm alignment in the flexion-extension and pronation-supination directions changed in the painful Experimental block. Similarly, average vector length was greater for epoch 5 of the *Pain 5–0* group than epoch 5 of the *Control g*roup (post-hoc: p = 0.03), and average vector length was greater in epoch 5 than epochs 1–3 (post-hoc: all p<0.02) during *Pain 5–0*.

### Was the movement option that induced no/less noxious input used more often during the painful experimental blocks?

During the painful Experimental blocks of the painful groups, the solution that was determined by the experimental paradigm to induce less/no noxious input was experienced for at least one repetition of the task by 19/21 participants in the *Pain 5–1* group, and 6/6 in the *Pain 5–0* group. However, contrary to hypothesis (iv), this less/non-painful movement solution was not systematically used more frequently during the painful Experimental block than Baseline block in either *Pain 5–1* (Baseline: 27(4)%; Experimental: 21(10)%) or *Pain 5–0* (Baseline: 29(11)%, Experimental: 31(17)%) groups (Main effects—Group: F_1,25_ = 0.95, p = 0.34, η_p_^2^ = 0.04; Block: F_1,25_ = 0.18, p = 0.67, η_p_^2^ = 0.01; Interaction—Group×Block: F_1,25_ = 0.47, p = 0.50, η_p_^2^ = 0.02).

### Did movement variability increase during pain in a manner consistent with a search for a new movement solution?

**Fig 5:**
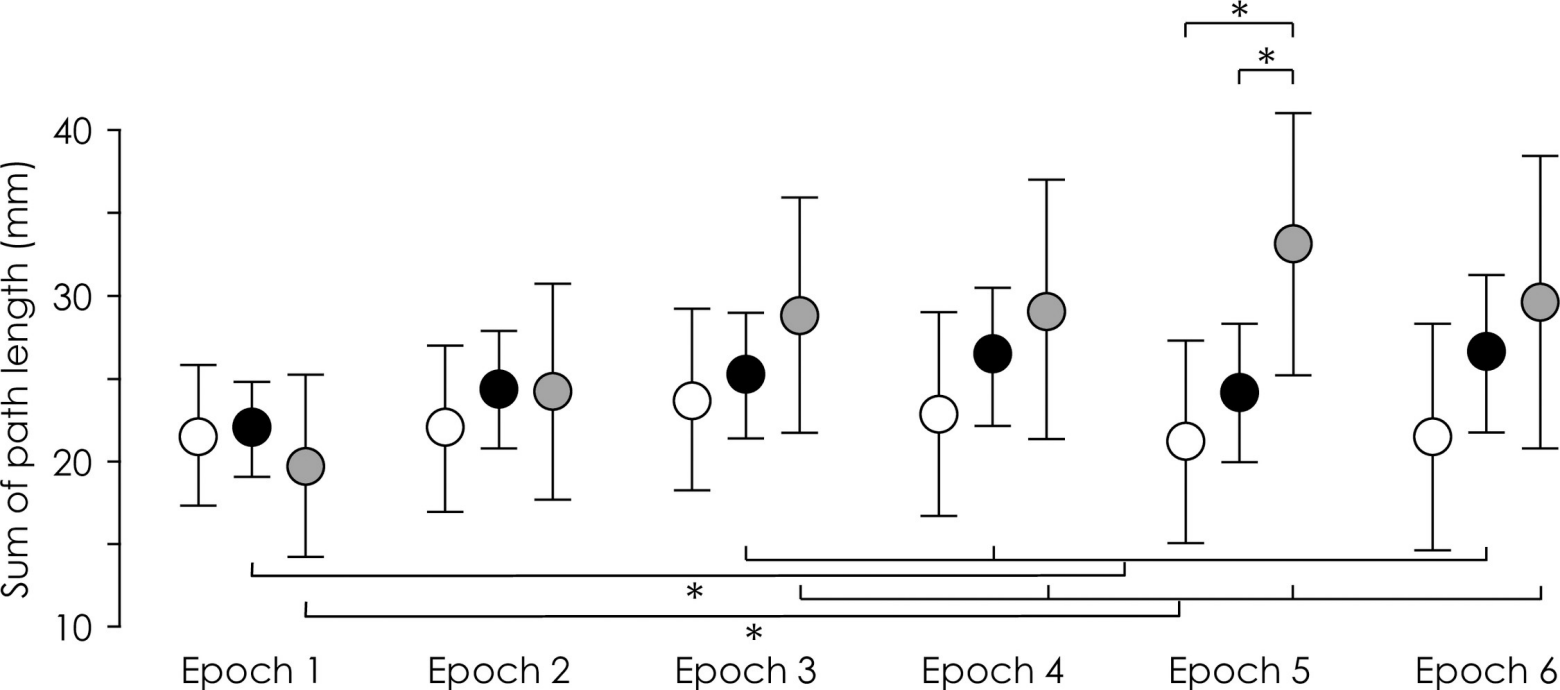
Group data for movement variability, quantified as with sum of path length during averaged across blocks. Group mean and 95% CI of sum of path length of 10-repetition epochs for the *Control* (white), *Pain 5–1* (black), and *Pain 5–0* (grey) groups. *—p<0.05 between bracketed items.

During the painful Experimental blocks, movement variability, measured as sum of path length, was greater during the middle/end of the block than the start (epoch 1) (Interaction—Group×Epoch: F_10,170_ = 2.42, p = 0.01, η_p_^2^ = 0.12; *Pain 5–1* epochs 3, 4, 6: post-hoc: all p<0.025; *Pain 5–0* epochs 3–6 post-hoc: all p<0.001; *Control* post hoc: all P>0.20; Fig 5), but this was apparent for both Baseline (painfree) and Experimental (painful) blocks (Interaction—Group×Block×Epoch: F_10,170_ = 0.65, p = 0.77, η_p_^2^ = 0. 04). Although the sum of path length was greater in *Pain 5–0* than *Pain 5–1* and *Control* for epoch 5 (post-hoc: p = 0.035) again, this was not specific to the painful Experimental block. That is, contrary to hypothesis (v), movement variability was not systematically greater in the presence of pain (Experimental block) than during the painless Baseline block.

### Was there individual variation in the strategy used to adapt to noxious input?

**Fig 6:**
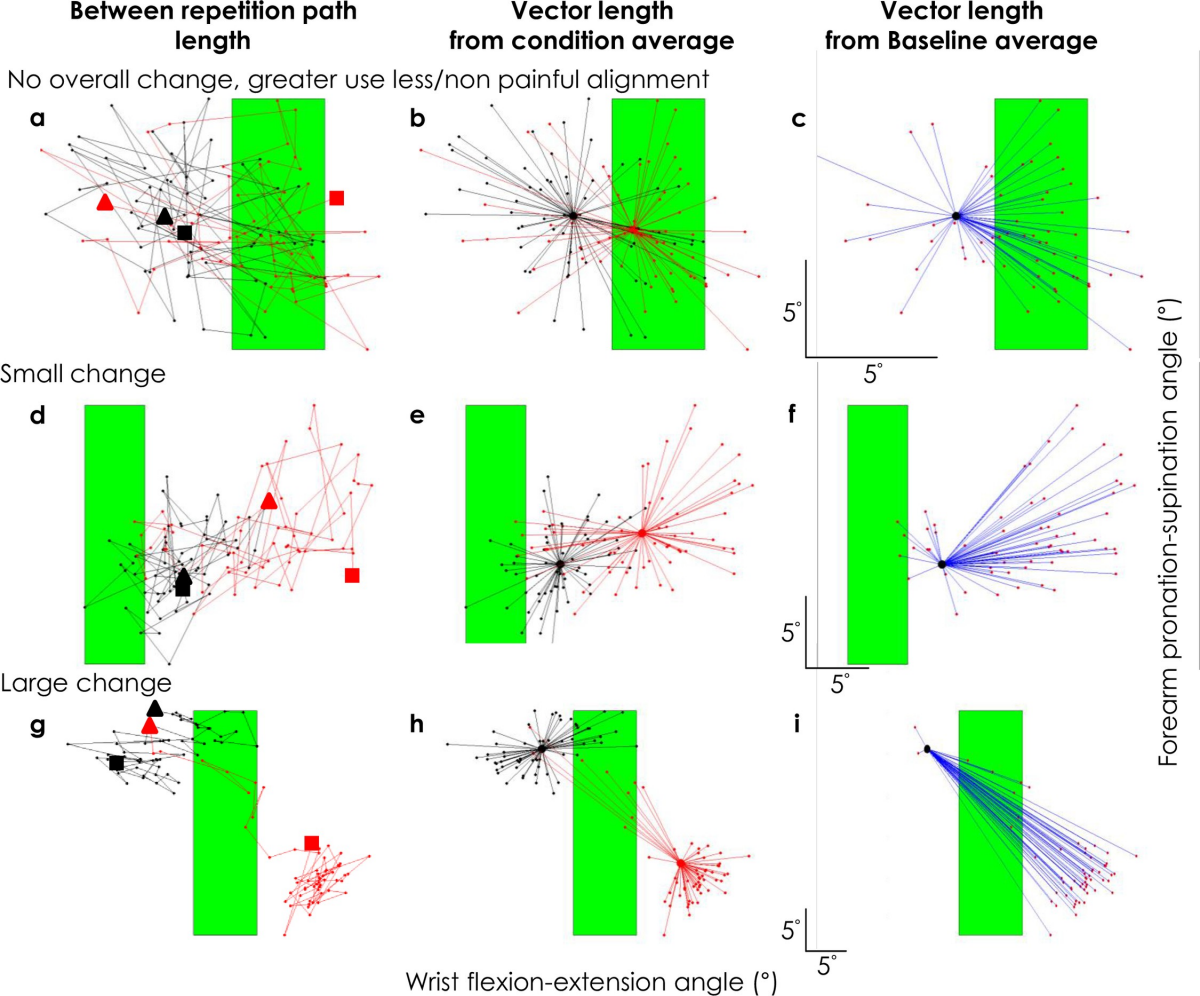
Two-dimensional movement maps that depict the three distinct movement strategies used by three separate representative participants in the *Pain 5–1* and *Pain 5–0* groups who used ‘no overall change, greater use of less noxious stimulation’ (a-c), ‘small change’ (d-f), or ‘large change’ (g-i). In all plots black and red circles/lines depict the Baseline and Experimental blocks, respectively. Plots show forearm pronation-supination angle vs. flexion-extension angle for the 60 repetitions of each block. Separate sub-plots represent different analyses. In the left panels, lines show path length plotted between consecutive repetitions of each block starting with repetition 1 (large triangles) and ending with repetition 60 (large squares). Middle panels show the alignment of the wrist/forearm in the secondary planes for the 60 repetitions relative to the mean alignment (large circles) for each block. Right panels show ‘vector length’ (blue lines) of wrist/forearm alignment in the pain condition relative to the mean alignment of the Baseline block. Note that different scales are used for the axes of the three movement strategies. Scale bars in the bottom left corner of each sub-plot in the right panels represents 5° in each direction (i.e. pronation-supination, flexion-extension). Green shaded areas represent the range of alignments in the flexion-extension direction selected for the less intense noxious stimulus. The three plots for each participant are presented with the same scale.

Visual inspection of our data revealed that individual participants appeared to use different strategies to adapt movement during pain. On this basis we undertook additional exploratory analysis (Table 1). Our major consideration was how the position of the wrist/forearm in the flexion-extension and pronation-supination directions was modified during the painful Experimental block relative to the Baseline block as they attempted (not always successfully) to maintain achievement of the goal in the radial-ulnar deviation direction. Plots of alignment of the wrist/forearm in the secondary planes (flexion-extension vs. pronation-supination; “alignment map”) as the wrist crossed radial-ulnar neutral revealed three distinct patterns of adaptation (Fig 6). Strategy 1 (no change) involved no difference in vector length (i.e., wrist/forearm movement in the same region of the alignment map during the non-painful Baseline and painful Experimental blocks with little overall change in the wrist/forearm alignment in the secondary planes relative to the mean of the Baseline block (shown as vector length in Fig 6C). Strategy 2 (slow small change) involved initial movement in the same map region during the painful condition followed by gradual movement to a new distinct region with small advances with each repetition (Fig 6F). Strategy 3 (rapid large change) involved a large change in secondary plane alignment to a different map region on the first or second repetition of the painful Experimental block (Fig 6I).

**Table 1:** Data for individual participants for each strategy group.

Exp	Strat	Vector length			Pain		Reps in no/low pain region	Reps to no/low pain region	Path length	
		Base: Average	Pain: Average	Ratio: Pain/Base	First 20 epochs: Average	Last 20 epochs: Average	Base: %	Pain: %	Base: mean epoch 1–2	Pain: mean epoch 1–2
Exp 5–1	1	4.5	5.0	1.1	5.0	4.0	35.0%	23.3%	35.6	37.2
Exp 5–1	1	3.4	4.1	1.2	2.0	0.5	31.7%	56.7%	30.0	29.7
Exp 5–1	1	3.0	4.1	1.4	4.0	4.0	18.3%	30.0%	19.0	19.6
Exp 5–1	1	2.6	3.8	1.5	4.0	1.0	28.3%	20.0%	15.0	22.9
Exp 5–1	1	3.1	3.3	1.0	7.0	4.0	26.7%	26.7%	27.3	21.9
Exp 5–1	1	3.5	4.8	1.4	5.0	4.0	40.0%	91.7%	19.6	18.1
Exp 5–1	1	2.5	3.0	1.2	4.0	2.0	45.0%	8.3%	25.4	17.4
Exp 5–1	1	3.0	4.2	1.4	6.0	5.0	11.7%	38.3%	24.3	24.7
Exp 5–0	1	3.2	4.4	1.4	4.0	0.0	30.0%	48.3%	23.0	24.2
Exp 5–0	1	2.9	2.8	1.0	4.0	4.0	20.0%	25.0%	17.7	19.1
Exp 5–1	2	4.0	11.7	2.9	4.0	4.5	26.7%	31.7%	30.3	23.2
Exp 5–1	2	4.7	13.6	2.9	2.0	1.0	23.3%	13.3%	18.7	28.8
Exp 5–1	2	2.4	5.8	2.4	1.0	1.0	25.0%	18.3%	19.8	25.8
Exp 5–1	2	3.1	8.7	2.8	5.0	4.0	21.7%	3.3%	22.0	29.9
Exp 5–1	2	3.9	6.2	1.6	5.0	2.0	25.0%	3.3%	15.9	22.9
Exp 5–1	2	2.7	9.4	3.5	4.0	2.0	46.7%	1.7%	24.6	23.2
Exp 5–1	2	3.3	11.0	3.3	6.0	2.0	8.3%	16.7%	20.3	41.7
Exp 5–1	2	3.5	5.5	1.5	5.0	4.0	28.3%	45.0%	17.9	13.8
Exp 5–1	2	3.5	8.2	2.4	4.0	4.0	16.7%	5.0%	31.1	33.7
Exp 5–0	2	2.8	4.9	1.7	4.0	6.0	38.3%	41.7%	15.2	13.6
Exp 5–0	2	2.7	7.0	2.6	4.0	3.0	8.3%	10.0%	11.2	16.5
Exp 5–0	2	4.8	8.7	1.8	3.0	4.0	31.7%	56.7%	35.6	31.8
Exp 5–1	3	4.5	20.8	4.6	5.0	5.0	25.0%	10.0%	19.9	32.6
Exp 5–1	3	1.9	10.1	5.3	2.0	1.0	38.3%	3.3%	15.3	32.7
Exp 5–1	3	1.3	10.0	7.7	7.0	7.0	28.3%	0.0%	8.7	17.7
Exp 5–1	3	0.8	4.4	5.6	4.0	4.5	26.7%	0.0%	6.3	7.0
Exp 5–0	3	2.7	17.2	6.3	7.0	5.0	48.3%	3.3%	18.9	36.7
Mean	1	3.2	4.0	1.3	4.5	2.9	28.7%	36.8%	23.7	23.5
SD	1	0.6	0.7	0.2	1.4	1.8	10.1%	23.8%	6.2	6.1
Mean	2	3.5	8.4	2.5	3.9	3.1	25.0%	20.6%	21.9	25.4
SD	2	0.8	2.7	0.7	1.4	1.5	11.1%	18.7%	7.3	8.4
Mean	3	2.2	12.5	5.9	5.0	4.5	33.3%	3.3%	13.8	25.3
SD	3	1.5	6.5	1.2	2.1	2.2	9.9%	4.1%	6.1	12.5

Strategy 1—no change; Strategy 2—slow small change; Strategy 3—rapid large change; Exp–experiment; Strat–strategy.

**Fig 7:**
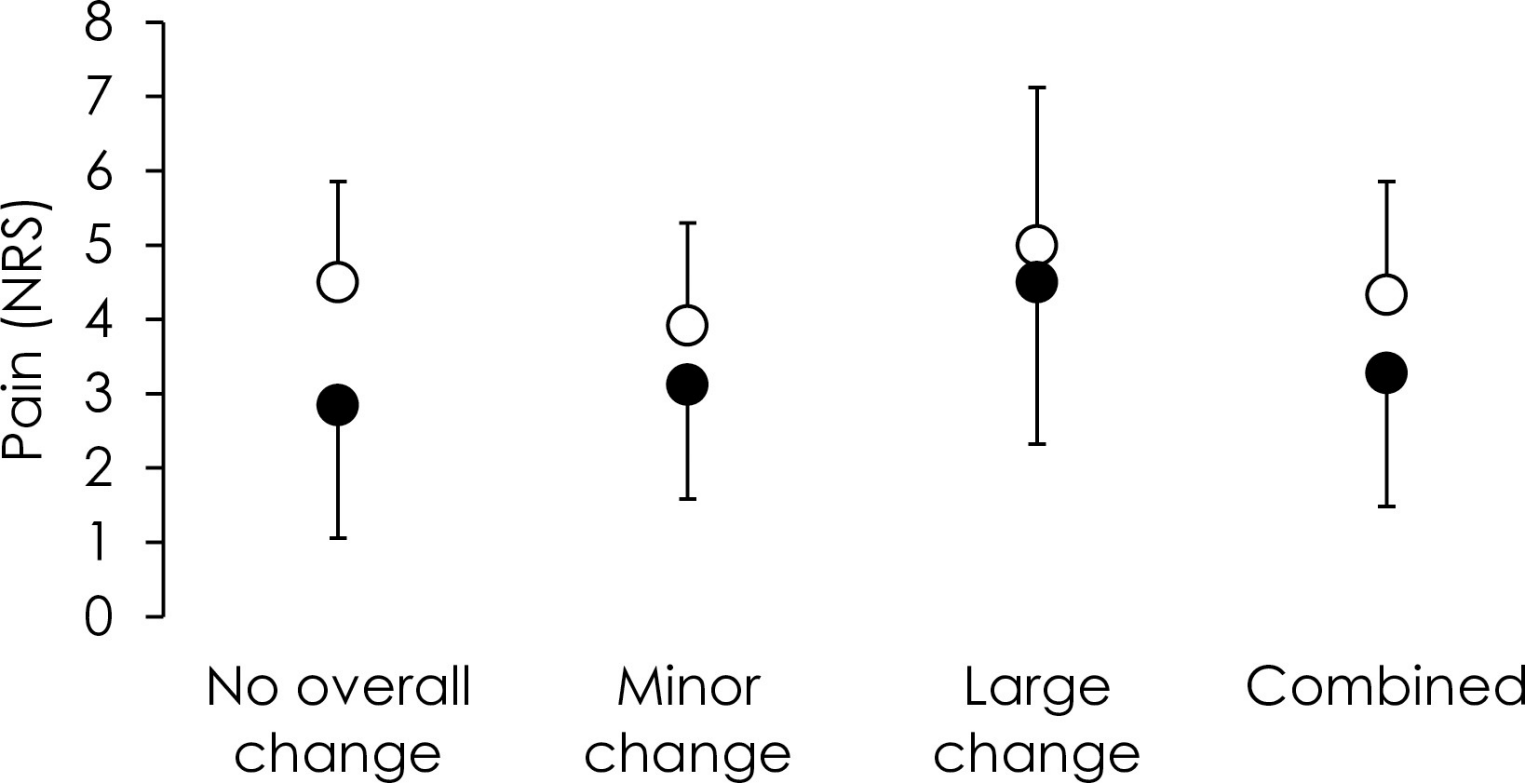
Pain reported at the start (first 20 epochs; open circles) and end (last 20 epochs; closed circles) for each strategy and with all strategies combined. Each strategy led to a reduction in pain, although only the “no change” strategy involved greater use of the range with reduced noxious input. The reduction in pain in the “small” and “large” change strategy was achieved despite no change in noxious input.

To quantify each strategy the ratio of average vector length for the Experimental vs. Baseline blocks was calculated. Strategy 1 (no change) was defined as an Experimental-Baseline ratio of <1.5; Strategy 2 (slow small change) as a ratio of 1.5–4; and Strategy 3 (rapid large change) as a ratio >4. In *Pain 5–1*, 8 participants used Strategy 1, 9 participants used Strategy 2 and, and 4 used Strategy 3. In *Pain 5–0*, the proportions were 2/6, 3/6 and 1/6, respectively. As expected, the ratio was <1.5 (i.e., no change) for all participants in the painfree *Control* group. Regardless of strategy, all participants experienced reduced pain by the end of the block (repeated measures ANOVA (Strategy (1 vs. 2 vs. 3) x Time (start [first 20 epochs] vs. end [last 20 epochs]); main effect Time; F_1,24_ = 10.68, p = 0.003, η_p_^2^ = 0.31; interaction Time x Strategy; F_2,24_ = 1.40, p = 0.27, η_p_^2^ = 0.10) (Fig 7).

Comparison of features of movement strategy between these subgroups revealed distinct solutions to the problem of selection of a new movement solution during pain. First, although participants who adopted Strategy 1 did not change the average vector length, they used the experimentally-determined region with less/no noxious stimulation more frequently than participants who had a large change in vector length (Strategy 3) (one-way ANOVA (Strategy 1 vs. 2 vs. 3); F_2,24_ = 5.21, p = 0.013, η_p_^2^ = 0.30; post-hoc: p = 0.004) and a similar non-significant tendency relative to those who used Strategy 2 (post-hoc: p = 0.062). Second, participants who adopted Strategy 3 had a greater initial sum of path length (i.e. greater variation in secondary plane) (quantified as mean epoch 1 and 2) of the Experimental (25.3(7.9)deg) than Baseline (13.8(6.2)deg) block (repeated-measures ANOVA; Block (Experimental vs. Baseline; repeated measure); Strategy (1 vs. 2 vs. 3; between-subject factor; Interaction—Block×Strategy F_2,1_ = 5.25 P = 0.013, η_p_^2^ = 0.30; post hoc: P = 0.0002). An additional observation was that the group who adopted Strategy 3 had lower initial sum of path length (Baseline Strategy 3 = 13.8(6.2)deg) than the participants who adopted the other Strategies (Baseline Strategy 1 = 23.7(4.4)deg; Strategy 2 = 21.9(4.0)deg)(both: post hoc: P>0.05). The frequency of exposure to the target region with less noxious input was similar for all Strategy subgroups (Strategy 1–29(10)%; Strategy 2–25(11)%; Strategy 1–33(10)%; one-way ANOVA (Strategy 1 vs. 2 vs. 3); F_2,24_ = 1.15, p = 0.33, η_p_^2^ = 0.09).

## Discussion

The results of this study provide novel observations and new understanding of the movement adaptation to pain. We show that movement adapts when it provokes a noxious input, this adaptation is accompanied by reduced experience of pain, but this does not depend on whether the new movement solution provokes less noxious input. Our experimental paradigm provided the possibility to complete the task with less/no nociceptive stimulation, and almost all participants were exposed to this benefit. However, this strategy for pain reduction was only selected by 37% (10/27) of participants. Instead, participants used various solutions that differed from their natural strategy and achieved a pain reduction, regardless of whether or not the intensity of applied noxious input changed. One group responded to pain with a larger change in strategy and initially increased between-repetition variation, consistent with a search for a new solution, but there was no increase for others. Taken together these observations imply individuals select different solutions to reduce pain, and that for many, “taking action” to reduce threat is perhaps more important than an “actual” reduction of nociceptive afferent activity.

### Methodological considerations

Interpretation of the present results require consideration of several methodological issues. First, although we tested for habituation of the noxious stimuli, this was done in a slightly different context to the experimental tests. Although habituation is reported for longer duration stimulation [23], this was not apparent with our protocol of brief intermittent stimuli. Second, motion of the hand was measured with respect to the external reference frame and interpreted as movement relative to the forearm as that segment was stabilised. Although limited possible motion of the forearm may introduce small errors in the estimate of wrist angle this would not be likely to affect the main finding of the study. Third, the noxious stimulation paradigm was dissimilar to clinical pain in several respects which may have impacted the movement adaptation. Notably the noxious stimulus reduced in a step manner rather than gradual change that might be expected naturally and the provocation of pain either side of the target region. Further, if participants had greater exposure to the no/low pain region they may have been more likely to use that range. Modification of our instructions so that participants were not completely naïve to the presence of a no/low pain option may have led to different results. Fourth, a larger sample size might provide greater confidence in the non-significant differences.

### Why did experimental pain compromise the task goal?

The target radial deviation angle was achieved less often (~10% decrease) during pain than *Control* blocks. This concurs with observations for some [24–26] but not all previous studies [10, 27]. Three possibilities might explain this difference. First, pain modality may be relevant. In an earlier study, performance of radial-ulnar deviation, as used here, was maintained during pain induced by hypertonic saline injection [10], which induces tonic pain unrelated to movement. Movement-related pain, such as that used here, is potentially more disruptive and this might be mediated by distraction.

Second, emphasis placed on goal attainment differs between studies. Ingham et al. [27] emphasised task accuracy and it was not reduced with pain. In the present study the target was indicated but not emphasised. Third, perceived cost/benefit of goal attainment differs between studies. Accuracy is less in studies where goal attainment provokes pain (tongue force against a pad coated with capsaicin [24]), but maintained when goal attainment is encouraged by provision of a reward (monetary) or penalty (painful electric stimuli) for accurate and inaccurate pointing to a target [28]. In the absence of explicit benefit/cost our participants may have lacked motivation to maintain the task goal.

### Was a new movement solution used during pain?

All participants adopted a new movement solution during pain. When painful electrical stimuli were applied throughout the Experimental blocks, group data show that wrist/forearm alignment changed relative to Baseline, and this was accompanied by reduced pain. Our exploratory analysis of individual data showed three patterns of movement adaptation in the secondary planes: a large initial alignment change, a progressive smaller alignment change, or maintenance of movement within the baseline range but with greater use of the ranges that was associated with less/no pain. Despite exposure to the non/less painful alternative, most participants (63%) did not select this movement strategy. Instead, they adopted another solution, but still experienced a pain reduction.

There are several possible explanations why the option with reduced noxious stimulus was not selected. First, to maintain the experimentally provided solution, it would be necessary for participants to “realize” that noxious input could be reduced, and which movement component determined the stimulation intensity. Participants were not informed that stimulus intensity could be modified by movement strategy. Earlier work shows participants change movement strategy if they are aware of the task manipulation (reduced efficiency of one limb in a bilateral task [29]), but not if the manipulation is applied without their knowledge [18]. Thus, despite exposure to the benefit (reduced noxious input) of the experimentally provided solution, failure to interpret the relationship between movement and noxious input might explain its non-use. Further, the relationship between movement and noxious input may not have been intuitive. Other studies with simpler solutions to reduce pain have successfully adapted movement strategy (e.g. reduced movement amplitude when pain is provoked at the end-point of a pointing task [28].

Second, factors other than pain might be considered for strategy selection. Although it is assumed reduced noxious input is prioritised, results of this study and others [7, 9] suggest otherwise. For instance, noxious input to the infrapatellar fat pad with hypertonic saline modifies knee extension force angle, but not always in a direction expected to reduce load on the irritated tissue [9]. Further, measures of muscle activation (electromyography) and muscle stress (elastography) during pain (hypertonic saline) show no systematic change for tasks with few degrees of freedom (elements), but changes during more complex tasks [30]. These observations can be explained if reduced nociceptive input is not the sole consideration and strategy selection involves balancing this with other factors such as optimization of end-point error [31], energy expenditure [18, 32], and muscle force [30, 33]. These factors could vary between individuals because of anatomical differences (e.g. relative muscle force generating capacity [34]). There are numerous examples where movement is adapted to meet other goals. In non-painful situations energy consumption is minimised to ensure muscles can meet energy requirements of subsequent movements [35, 36] and the “*minimum variance model*” predicts that the nervous system activates muscles in a manner that minimizes end-point error [22]. It is unclear how these factors are balanced, or whether their relative importance is altered in pain/injury.

Finally, the perceived *benefit* of an adaptation might not depend on an actual reduction of noxious input, but instead depend on having “taken action” (see below). The selected solution may be uncoupled from a relationship to the noxious input.

### Was movement variability used to find a new movement solution during pain?

It has been proposed that the nervous system takes advantage of variation in the elements of movement to find a less provocative movement solution, and once identified, uses this solution more frequently to reduce pain [12]. Consistent with this hypothesis, studies of multi-joint tasks identified increased movement variability [11, 12] preceding resolution to a modified movement strategy. In the present study, movement variability (sum of path length) was not increased systematically. One subgroup of participants (18.5%; 5/27) increased movement variability after pain onset, and this group made a large change in alignment of the wrist/forearm in the secondary plane. Other participants gradually moved away from the initial strategy or remained within the initial range but with greater use of the experimentally provided less/non painful option, and did so without any significant increase in change of alignment between successive repetitions. Together the data support the hypothesis of a search for a less painful movement strategy during pain [1, 11, 12], but increased movement variability was not necessarily used for this search. It is possible that this simple movement with few degrees of freedom have limited scope to increase movement variability.

### Pain relief did not depend on adoption of a strategy that reduced noxious input

By design, the experimentally provided solution for pain reduction was within the range of secondary plane alignments used in the Baseline block. Although almost all participants used this alignment for at least one repetition, only 37% used it more frequently and received reduced or no noxious stimulation. Regardless, all experienced less pain in their adapted movement strategy. This observation can be explained by the physiology of pain and provides novel insight into the purpose and selection of adapted movement strategies during noxious stimulation.

Pain relates to nociceptive input in a manner that is not straightforward. This is because pain is an output generated by the brain that depends not only on nociceptive input [16], but features including; cognitive interpretation (e.g. threat, fear), processes that sensitise the nervous system (peripherally, centrally) to nociceptive input [37], and descending modulatory pathways (facilitatory, inhibitory) that influence spinal nociceptive processing [38]. The present data concur with the proposal that pain motivates an individual to “take action” to reduce threat to the tissues, and that the act of “taking action” is sufficient to reduce the experience of pain [17]. Our observed pain reduction in the absence of diminution of the noxious stimulus for 63% of participants, and without evidence of habituation to the stimulation, supports this proposal. This observation highlights the complexity of interpretation of the relationship between pain and movement. Although movement may excite nociceptive afferents, if pain is increased or decreased by a change to movement strategy, this cannot be easily interpreted as greater or lesser threat to the tissues.
